# Clinical and epidemiological aspects of chronic Chagas disease from Southern Brazil

**DOI:** 10.1590/0037-8682-0225-2020

**Published:** 2020-10-21

**Authors:** Kárita Cláudia Freitas Lidani, Thaisa Lucas Sandri, Ricardo Castillo-Neyra, Fabiana Antunes Andrade, Cesar Maistro Guimarães, Eduardo Nunes Marques, Marcia Holsbach Beltrame, Robert Hugh Gilman, Iara de Messias-Reason

**Affiliations:** 1 Universidade Federal do Paraná, Departamento de Patologia Médica, Hospital de Clínicas, Curitiba, PR, Brasil.; 2 University of Tübingen, Institute of Tropical Medicine, Tübingen, BW, Germany.; 3 University of Pennsylvania, Perelman School of Medicine, Department of Biostatistics, Epidemiology & Informatics, Philadelphia, PA, USA.; 4 Universidade Federal do Paraná, Hospital de Clínicas, Unidade de Terapia Intensiva, Curitiba, PR, Brasil.; 5 Universidade Federal do Paraná, Departamento de Genética, Laboratório de Genética Molecular Humana, Curitiba, PR, Brasil.; 6 Johns Hopkins Bloomberg School of Public Health, Department of International Health, Baltimore, MD, USA.

**Keywords:** Chagas disease, *Trypanosoma cruzi* infection, Cardiomyopathy, Epidemiology

## Abstract

**INTRODUCTION:**

Patients with Chagas disease (CD), caused by *Trypanosoma cruzi*, present a higher risk of developing other chronic diseases, which may contribute to CD severity. Since CD is underreported in the southern state of Paraná, Brazil, we aimed to characterize clinical and epidemiological aspects of individuals chronically infected with *T. cruzi* in Southern Brazil.

**METHODS:**

A community hospital-based study was performed, recording clinical/demographic characteristics of 237 patients with CD from Southern Brazil. To estimate the association between different forms of CD and sociodemographic and clinical variables, multiple logistic regression models were built using the Akaike information criterion.

**RESULTS:**

Mean age was 57.5 years and 59% were females. Most patients’ (60%) place of origin/birth was within Paraná and they were admitted to the CD outpatient clinic after presenting with cardiac/digestive symptoms (64%). The predominant form of CD was cardiac (53%), followed by indeterminate (36%), and digestive (11%). The main electrocardiographic changes were in the right bundle branch block (39%) and left anterior fascicular block (32%). The average number of comorbidities per patient was 3.9±2.3; systemic arterial hypertension was most common (64%), followed by dyslipidemia (34%) and diabetes (19%); overlapping comorbidities were counted separately. Male sex was associated with symptomatic cardiac CD (OR=2.92; 95%CI: 1.05-8.12; p=0.040).

**CONCLUSIONS:**

This study provided greater understanding of the distribution and clinical profile of CD patients in Southern Brazil, indicating a high prevalence of comorbidities among these patients who are a vulnerable group due to advanced age and substantial risk of morbidity.

## INTRODUCTION

Chagas disease (CD) is a neglected tropical disease caused by the protozoan *Trypanosoma cruzi* which leads to higher rates of morbidity and mortality in Latin America than any other parasitic disease, resulting in significant decreases in the quality of life due to disability^1^. Although most infected individuals remain asymptomatic for their entire lives, about 20-30% develop chronic Chagas cardiomyopathy (CCC)^2^ ^,^ ^3^. CCC has a wide range of manifestations, including arrhythmias, heart blocks, heart failure, thromboembolism, stroke and sudden death^4^ ^,^ ^5^. 

All chronic CD forms present a high prevalence of comorbidities, including systemic arterial hypertension (SAH), dyslipidemia, and diabetes mellitus^6^ ^-^ ^11^. CD is also a major cause of cardioembolic strokes, which is twice as common in CCC as in other types of cardiomyopathy^12^ ^,^ ^13^. Sudden death is considered the main cause of death in patients with CD, followed by refractory heart failure and thromboembolism^14^. Since some risk factors such as obesity, smoking, and SAH are associated with the development of cardiac and cerebrovascular diseases, they may also negatively impact the quality of life and prognosis of individuals with chronic CD.

Globally, the annual burden from infected individuals is $627.46 million in health-care costs and $806,170 disability-adjusted life-years (DALYs). Ten percent of these costs come from the United States^15^. In Brazil, the highest prevalence of CD is in the Northeastern and Southeastern regions^16^. In contrast, only two studies of CD have been done in the state of Paraná (Southern Brazil) and these were from the Central-North area^17^ ^,^ ^18^. Moreover, in general, the distribution of the chronic cases of CD is silent and the numbers are underestimated due to the lack of surveillance and notification. In this context, we aimed to characterize epidemiological and clinical aspects of individuals chronically infected with *T. cruzi* in Southern Brazil.

## METHODS

### Ethical statement

This study was approved by the Ethics Committee of the Clinical Hospital of the Federal University of Paraná, Curitiba (HC/UFPR) (n. 360.918/2013-08). According to the Declaration of Helsinki, all subjects provided written and informed consent to participate in the study. 

### Study design

This is a cross-sectional and hospital convenience-based study carried out between June 2007 and June 2008, involving patients with chronic CD visiting the Hospital de Clínicas - Universidade Federal do Paraná - HC/UFPR (Federal University of Paraná), Southern Brazil, a reference center for chronic CD follow-up within the Sistema Único de Saúde (SUS, the Brazilian Unified Health System).

### Participant recruitment

Since SUS is an integrated medical care system, CD patients were referred to the HC/UFPR from several sources, such as blood banks (*T. cruzi* seropositive blood donors), infectious diseases clinics, or outpatient clinics that identified any cardiac and/or digestive symptoms related to CD at public hospitals in the state of Paraná. 

### Study population

A convenience sample of 237 patients with a diagnosis of *T. cruzi* infection, born (or resided during childhood) in endemic areas and aged eighteen years or over, were consecutively enrolled in this study. Pregnant women and patients with congenital, hypertensive or other associated cardiomyopathy were excluded from this study, as were individuals who were not available for consultation or whose medical records presented insufficient data. 

### Laboratory and clinical evaluation

Positive serology for anti-*T. cruzi* antibodies was based on Chemiluminescence Microparticle Immunoassay with 100% sensitivity (95% CI: 97.90 to 100%) and 99.93% specificity (95% CI: 99.80 to 99.99%) (Architect Plus Chagas, Abbott, USA)^19^, and indirect immunofluorescence with 100% sensitivity and specificity (IMUNO-Con Chagas, WAMA diagnóstica, Brazil)^20^ assay^21^.

The epidemiological, clinical, and nutritional profiles of individuals with chronic CD were evaluated. Examinations included blood count, blood glucose, and lipid profile. On the day of the medical consultation, socio-demographic information such as sex, age, predominant ancestry, smoking, alcohol consumption (measured as yes or no), weight, height, body mass index (BMI: weight (kg)/ [height (m)])2, obesity (BMI ≥30), place of birth and residence, diagnosis year, comorbidities, medication use, polypharmacy (≥5 medications) and CD-related symptoms, was obtained by interviewing the patients. Individuals who had smoked 100 or more cigarettes during their lives and were still smoking either daily or occasionally at the time of interview were considered tobacco smokers. For a quantitative measurement of smoking, a pack-years index was used (number of packs smoked per day multiplied by the years as a smoker, considering 20 cigarettes/pack)^22^. Alcohol consumption was defined as alcohol use on three days of the week for at least one year^23^ **.** Hypertension was deﬁned as a systolic blood pressure ≥140 mm Hg or diastolic blood pressure ≥90 mm Hg^24^, and diabetes mellitus as a fasting plasma glucose ≥126 mg/dL or patients who were under treatment with an oral antidiabetic agent or insulin^25^. Dyslipidemia was defined according to lipid profile and included serum levels of triglycerides, total cholesterol and fractions, following the V Brazilian Consensus on Dyslipidemia guidelines^26^. Systemic autoimmune diseases (SAD) included rheumatoid arthritis, systemic lupus erythematosus (and subsets of Lupus), Sjögren’s syndrome, systemic sclerosis, polymyositis, and dermatomyositis; all cases met the most current diagnostic criteria and were confirmed by a rheumatologist.

### Clinical forms of chronic Chagas disease

The definitions of clinical manifestations followed the criteria of the II Brazilian Consensus on Chagas Disease^21^ that included chest radiography (Proteus XR/a, GE), a 12-lead electrocardiogram (ECG), a transthoracic echocardiogram (ECHO [Hewlett-Packard, Sonos 5500, 2-4 MHz]), esophagography, and a barium enema (Siemens, AXIOM Iconos MD, Fluorospot Compact VE22). 

All ECG recordings were performed after a minimum of 5 minutes of inactivity and lasted from 10 to 30 seconds. Two well-trained physicians read all ECG recordings, and in case of disagreement between the two readings, a final decision was resolved by mutual consensus. All patients underwent a 2D-transthoracic echocardiogram using high-quality commercially available ultrasound systems. M-mode measurements were used to obtain left ventricular, aortic, and left atrial dimensions. The biplane Simpson’s method was applied for the calculation of the ejection fraction (EF), and wall motion was assessed in the following views: parasternal short-axis and the apical two-chamber, four-chamber, and long-axis. Normal reference ranges were based on the guidelines of the American Association of Echocardiography^27^.

To characterize the clinical form of the disease, the following criteria were used to define cardiac stages according to Acquatella^28^, based on a slight modification of published methods: (A) asymptomatic, normal ECG; (B) asymptomatic, characteristic ECG changes; (C) mild to moderate systolic dysfunction (ejection fraction (EF) 40-54%) and/or left ventricular dilatation, New York Heart Association (NYHA) class II; (D) severe systolic dysfunction (left ventricular end diastolic diameter >57mm, EF <40%, NYHA classification III or IV). Chronic asymptomatic individuals (stage A), also called indeterminate form, present positive serological tests and/or are positive for the presence of *T. cruzi* in parasitological examination but do not present clinical manifestations related to CD and have no abnormalities in the ECG, radiological study of chest, esophagus and colon^29^. CCC was defined as an electrocardiographic test suggestive of cardiac involvement in *T. cruzi*-infected patients, being either asymptomatic (stage B) or symptomatic (C + D stages)^28^. 

For the digestive form, the radiological exams (esophagography and barium enema) were analyzed through visual observation and sigmoid colon and rectum measurements^30^. This clinical form was defined as dilation of the gastrointestinal tract and gastrointestinal motor disorders, including megaesophagus and megacolon^31^. The digestive involvement was classified as either megaesophagus or megacolon, and the association between cardiac and digestive forms was considered the cardiodigestive form.

### Study area and georeferenced data

**FIGURE 1:**
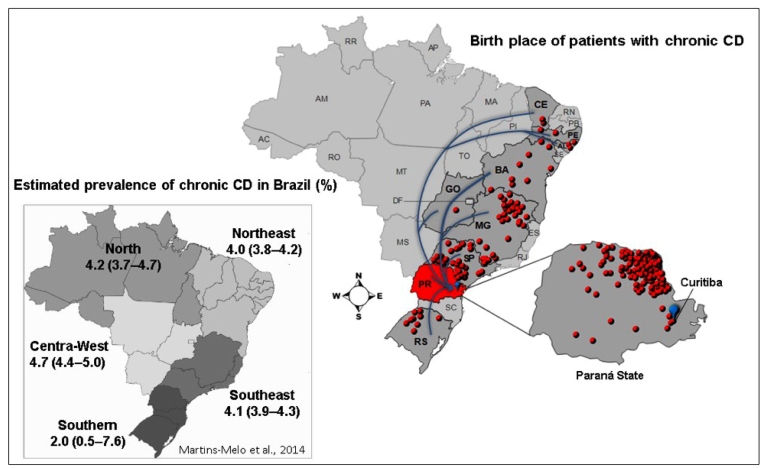
Place of origin of patients with Chagas disease (CD) attended in Curitiba (n = 237). Estimated prevalence (%) in Brazil per region according to Martins-Melo et al.^16^.

The outpatient clinic for CD from HC/UFPR is a reference center for disease management in the state of Paraná. The national pooled estimated prevalence for CD is 2.5% (95% CI: 2.3-2.6), and 2% for Paraná after the year 2000^16^. Data obtained regarding city of origin/birth and city of residence were transformed into geographical coordinates using the municipalities’ center, according to the Instituto Brasileiro de Geografia e Estatística (IBGE) (http://ibge.gov.br/cidadesat/xtras/home.php?lang=_EN) (Figure 1). 

### Data analysis

Sociodemographic data of the CD cases were compared among clinical forms. For bivariate analyses, chi-square and Fisher’s tests were used for frequency data (e.g. sex, ancestry group), and t-tests for continuous data (e.g. age) comparisons. A zero-inflated negative binomial model was used for the number of medications patients were taking. Among CD cases, variables statistically associated with the presence of cardiac involvement (B and C stages) were determined. Also, based on the experience of the research team, an inductive approach was used to determine different sets of relevant sociodemographic and clinical variables. Multiple logistic regressions models were built to test those sets and estimate the adjusted association between different forms of CD and those variables. Model selection was done using the Akaike information criterion (AIC), with p-values <0.05 considered statistically significant. Statistical analyses were performed using R software version 3.2 (R Foundation for Statistical Computing, Vienna, Austria - https://www.R-project.org)^32^.

## RESULTS

### Descriptive data

The case group consisted of 237 patients with chronic CD. The mean age was 57.5 years, with 40% of the sample considered elderly (≥60 years old); 59% were females, 77% were Euro-Brazilians, 17% were Afro-Brazilians, 4.6% were Amerindians, and 0.4% were Asian-Brazilians. The mean age of the asymptomatic A (n=85) and CCC (n=126, B+C+D) groups were 55.1 and 58.9 years, respectively (Table 1).

**TABLE 1:** Comparison of individual demographic and medical-related variables among patients with Chagas disease in univariate analysis.

Variable	Cases	A	B	C	D	B+C+D	A + B	C+D	A vs B	A vs C+D	A vs B+C+D	A+B vs C+D
	n=237	n=85	n=78	n=21	n=27	n=126	n=163	n=48	p-value	p-value	p-value	p-value
Age (average years)	57.52	55.12	56.74	65.86	59.56	58.87	55.90	62.31	0.257^§^	< 0.001^§^	0.005^§^	< 0.001^§^
Female N/total (%)	140/237	60/85	45/78	9/21	8/27	62/126	105/163	17/48	0.120*	< 0.001*	0.003*	<
	(59)	(71)	(58)	(43)	(37)	(49)	(64)	(35)				0.001*
BMI (average)	26.23	27.90	25.84	22.31	24.72	25.39	26.86	24.22	0.093^§^	0.004^§^	0.025^§^	0.016^§^
Ancestry group N/total (%)									0.096*	0.007*	0.050*	0.044*
Euro-Brazilian	183/237	74/85	56/78	13/21	21/27	90/126	130/163	34/48				
	(77)	(87)	(72)	(62)	(78)	(71)	(80)	(71)				
Afro-Brazilian	42/237	8/85	15/78	8/21	6/27	29/126	23/163	14/48				
	(18)	(9)	(19)	(38)	(22)	(23)	(14)	(29)				
Asian-Brazilian	1/237		1/78	0.00	0.00	1/126	1/163	0.00				
	(0.4)	0.00	(1)			(1)	(0.5)					
Amerindians	11/237	3/85	6/78	0.00	0.00	6/126	9/163	0.00				
	(4.6)	(4)	(8)			(5)	(5.5)					
Smoking N/total (%)	64/125	19/42	20/42	6/9	11/18	37/69	39/84	17/27	0.99*	0.233*	0.509*	0.203*
	(51)	(45)	(48)	(67)	(61)	(54)	(46)	(63)				
Alcohol consumption N/total (%)	30/54	9/19	11/19	2/3	5/9	18/31	20/38	7/12	0.745*	0.821*	0.657*	0.989*
	(56)	(47)	(58)	(67)	(56)	(58)	(53)	(58)				
Systemic arterial hypertension N/total (%)	145/225	58/82	44/75	12/20	16/26	72/121	102/157	28/46	0.157*	0.345*	0.137*	0.738*
	(64)	(71)	(59)	(60)	(61)	(60)	(65)	(61)				
Diabetes mellitus N/total (%)	43/225	16/82	11/75	4/20	7/26	22/121	27/157	11/46	0.553*	0.719*	0.956*	0.417*
	(19)	(19)	(15)	(20)	(27)	(18)	(17)	(24)				
Dyslipidemia N/total (%)	77/225	31/82	26/75	7/20	4/26	37/121	57/157	11/46	0.809*	0.159*	0.358*	0.165*
	(34)	(38)	(35)	(35)	(15)	(31)	(36)	(24)				
Continuous medication N/total (%)	163/235	61/85	49/78	19/21	22/27	91/126	110/163	43/48	0.294*	0.039*	0.999*	0.007*
	(69)	(72)	(63)	(90)	(81)	(72)	(67)	(89)				
Number of medications (average)	2.04	1.58	1.87	3.66	4.40	2.70	1.72	4.07	0.006+	< 0.001+	< 0.001+	< 0.001+

**Note:** All the clinical forms were included in the cases group. The cardiodigestive form was included among patients with cardiomyopathy (B+C+D). P-values estimated with Student’s t-test (§), Chi-squared test (*), Fisher’s exact test (#), and zero-inflated negative binomial model (+). ** Any rheumatic complaint was evaluated by a rheumatologist. Clinical stages of Chagas disease were classified as A, B, C or D. **BMI:** body mass index.

Most patients (60%, 136/225) were born in the state of Paraná, followed by Minas Gerais (15%), São Paulo (13%), Bahia (4%), Rio Grande do Sul (4%), Pernambuco (1%), Alagoas (1%), Ceará (1%) and Goiás (1%). All the patients were residents of the state of Paraná, and most of them lived in Curitiba or its surroundings (92%) (Figure 1). A total of 36% (70/195) reported having relatives with CD (1 to 11 relatives). When asked how they were diagnosed with CD, the majority of patients (65%, 155/237) reported that the presentation of the cardiac and/or digestive symptoms led them to seek medical care; 17% reported diagnosis via blood donation; and 17% reported other reasons.

The cardiac form of CD was the most common (53% [126/237]: 95 patients with the cardiac form and 31 with cardiodigestive form [29% of megacolon, 39% of megaesophagus, and 32% of megacolon associated with megaesophagus]) followed by the indeterminate form or stage A (36%) and the digestive (11%) form without CCC. The digestive form comprised 42% (11/26) of megacolon, 35% of megaesophagus, and 23% of megacolon cases associated with megaesophagus. CCC patients were divided according to functional heart classification, with 62% (78/126), 17%, and 21% for B, C, and D stages, respectively. Also, 43% (85/200) of patients had electrocardiographic abnormalities, the most frequent being a right bundle branch block (RBBB) (39%, 38/85) and left anterior fascicular block (LAFB) (32%). The median left ventricular ejection fraction (LVEF) was 67% (stages A: 70%; B: 68%; C: 50%; and D: 35%). The presence of an apical aneurysm was observed in 11% (14/126) of patients with cardiomyopathy. A total of 22% (28/126) of patients had a cardiac pacemaker (C and D stages).

Comorbidities were observed in 95% (224/237) of the patients, with an average of 3.9 ± 2.3 (range, 1-13) comorbidities per patient. Among the comorbidities, systemic arterial hypertension (SAH) was the most prevalent (64%, 145/225), followed by dyslipidemia (34%) and obesity (21% [31/148], with 38% classified as overweight). The median BMI was higher in asymptomatic (A+B) CD patients than in symptomatic (C+D) patients, and higher in the asymptomatic A group than in the CCC group and digestive clinical form. Diabetes mellitus was present in 19% (43/225) of patients, and systemic autoimmune diseases (SAD) in 2% (4/224 [rheumatoid arthritis, n= 3; and scleroderma, n=1]) (Table 1). History of other infections were reported in 10% (23/225) of patients and the most common were tuberculosis (2%, 5/23) and hepatitis B virus (1%, 3/23). Neoplasia was reported in 16% (36/225) of the patients: prostate (22%) and skin (17%) cancers were most prevalent. In Table 2, the reported prevalence of comorbidities in patients with chronic CD from other cross-sectional studies are compared with our findings. 

**TABLE 2:** Studies of comorbidities and lifestyle often observed in patients with chronic Chagas disease (CD) in Brazil.

Comorbidities	%	N	Mean age	CD form	Reference	Population
**SAH**	64	225	57.2	all	Present study	Hospital
	67	97	67.0	all	^7^	convenience based
	60	563	69.3	all	^9^	
	57	90	67.0	all	^6^	Hospital-based
	51	168	60.8	all	^33^	Community-based
	39	61	66.0	all	^34^	Hospital-based
	34	100	46.7	all	^35^	Hospital-based
	33	101	60.9	all	^36^	Hospital-based
	21	2,497	43.5	all	^37^	Hospital-based
						Hospital-based
						Hospital-based
**Obesity**	21	148	57.2	all	Present study	Hospital
	73^#^	74	55.6	IND	^8^	convenience based
	66^#^	168	60.8	all	^33^	Hospital-based
						Hospital-based
**Dyslipidemia**	34	225	57.2	all	Present study	Hospital
	76	74	55.6	IND	^8^	convenience based
	74	66	49.6	all	^10^	Hospital-based
	46	168	60.8	all	^33^	Hospital-based
	32	97	67.0	all	^7^	Hospital-based
	20	90	67.0	all	^6^	Hospital-based
						Hospital-based
**Diabetes mellitus**	19	225	57.2	all	Present study	Hospital
	24	168	60.8	all	^33^	convenience based
	14	97	67.0	all	^7^	Hospital-based
	12	66	49.6	all	^10^	Hospital-based
	10	90	67.0	all	^6^	Hospital-based
	0.4	2,497	43.5	all	^37^	Hospital-based
						Hospital-based
**Smoking**	51	125	57.2	al	Present study	Hospital
	34	38	45.0	all	^38^	convenience based
	23	66	49.6	all	^10^	Community-based
	18	563	69.3	all	^9^	Hospital-based
	14	168	60.8	all	^33^	Community-based
						Hospital-based
**Alcohol consumption**	56	54	57.2	all	Present study	Hospital
	32	38	45.0	all	^38^	convenience based
	24	168	60.8	all	^33^	Community-based
	19	563	69.3	all	^9^	Hospital-based
	17	66	49.6	all	^10^	Community-based
						Hospital-based

**Note:** Patients with overlapping comorbidities were considered for each comorbidity and included in the respective prevalence. **IND:** indeterminate form; 1 patient without clinical form; and 2 patients without CCC classification. **SAH:** systemic arterial hypertension. Among all the studies just ^9^,^35^ and ^38^ compared comorbidities between Chagas disease (CD) patients and seronegative controls, with statistically significant difference only for SAH in one of them^34^. # overweight or obese; *the diagnosis criteria were cholesterol >240 mg/dL and triglycerides >200 mg/dL.

Regarding lifestyle, the prevalence of smoking among patients was 51%, with pack-year varying from 1 to 101.5 (mean, 31.9). Alcohol consumption was reported among 56% (30/54) of patients. Continuous medication use was observed among 69% (163/236) of patients, with 21% (35/163) reporting polypharmacy. The most utilized class of drugs were the angiotensin-converting enzyme inhibitors (49%, 80/163), followed by diuretic drugs (38%) and beta blockers (25%). Regarding antiparasitic treatment, only 25% (55/216) of chronic CD patients were treated with benznidazole. The prevalence of smoking and alcohol consumption did not differ significantly among clinical forms; however, polypharmacy was more prevalent in patients with CCC than asymptomatic A patients (p=0.001). 

Comparing A to stages B+C+D in the multivariate model, male patients had 2.53 higher odds of having advanced forms of CD (B+C+D), and patients with 1 higher unit of BMI had 7% lower odds of having advanced forms of CD (B+C+D). Comparing A+B to C+D in the multivariate model, male patients had 2.92 times higher odds of having advanced forms of CD (C+D). All these associations were statistically significant after adjusting for age and ancestry group (Table 3).

**TABLE 3:** Factors associated with advanced stages of Chagas disease, estimated with multiple logistic regression.

	A vs B + C + D stages			A + B vs C + D stages		
Variable	OR	95% CI	p	OR	95% CI	p
Age in years	1.04	1.00 - 1.09	0.083	1.05	1.00 - 1.11	0.066
Male	2.53	1.14 - 5.60	0.022	2.92	1.05 - 8.12	0.040
BMI	0.93	0.87 - 0.99	0.037	0.93	0.86 - 1.01	0.089
Ancestry group						
Euro-Brazilian	Ref.	Ref.	Ref.	Ref.	Ref.	Ref.
Afro-Brazilian	2.31	0.82 - 6.54	0.115	1.23	0.37 - 4.06	0.735
Asian-Brazilian	*	*	*	*	*	*
Amerindians	5.61	0.53 - 59.66	0.153	*	*	*

**Note:** All the comparisons were adjusted for age and ancestry group. **BMI:** body mass index; **CI:** confidence interval; **OR:** odds ratio. *inestimable odds ratios due to small sample size or lack of variability. Clinical stages of Chagas disease were classified as A, B, C or D.

## DISCUSSION

This is the first study to characterize the clinical and epidemiological aspects of chronic CD in patients in Curitiba, Southern Brazil. We found a high prevalence of comorbidities regardless of CD clinical forms, which may increase morbidity and mortality leading to a substantial demand for health services and a decrease in quality of life. It is important to consider such risk factors as part of a mandatory routine in the follow-up of these individuals, especially in men since they represent the majority of patients presenting with more severe clinical forms of CD. These findings may help to understand the main clinical features observed in patients with chronic CD and their influence on the pathogenesis of the disease.

SAH was the most prevalent concomitant disease in chronic CD patients (64% overall; 66% of these were considered elderly), while it affects 31-32.5% of the general population and over 60% of the elderly^39^ ^-^ ^41^. Our findings reflect those of a number of previous studies (51 to 67%)^6^ ^,^ ^7^ ^,^ ^9^ ^,^ ^11^ ^,^ ^33^, although some have found lower rates of SAH among CD patients (20 to 34%)^35^ ^-^ ^37^. Diabetes and dyslipidemia affected 19% and 34%, respectively, of our CD patients, and diabetes was similar to the proportion described in other reports (20%, 24%, and 14%)^7^ ^,^ ^11^ ^,^ ^33^. The proportion of dyslipidemic patients in our study was lower than previous reports (46 and 76%)^8^ ^,^ ^33^. These discordances could be due to several factors, including the intrinsic complexity of CD, the age of patients, different populations, diagnosis criteria, sensitivity and specificity of tests, and sample sizes used. 

The association between SAH and CD is controversial. It has been demonstrated that *T. cruzi* is able to induce microvascular lesions in the host mainly by the release of thromboxane A2 and endothelin-1, leading to vasoconstriction and platelet aggregation, which might result in hypertension and impairments in the systemic vasculature^42^ ^,^ ^43^. In addition, *T. cruzi* can infect adipose tissue and cause lipid and glucose metabolism imbalances^44^ ^-^ ^47^, with possible impact on cardiovascular diseases. 

Male sex has been associated with cardiac symptomatic forms. Male sex has been considered a poor prognosis factor in chronic CD^11^ ^,^ ^48^ ^-^ ^51^ and independently associated with reduced myocardial function mainly due to myocardial fibrosis^52^. In mouse models, the immune response against *T. cruzi* infection was less favorable in males and linked to gonadal hormone differences^53^ ^,^ ^54^. The long period of time necessary for the development of tissue damage in chronic CD^55^ ^,^ ^56^ may explain the trend for association between advanced aging and symptomatic forms. Significantly lower BMI in more advanced stages of CD (B+C+D) may be related to the possible role of cardiac natriuretic peptides in stimulating lipolysis^57^ during heart damage, besides the presence of wasting syndrome and malnutrition often observed in patients with heart failure, in which calorie and protein intake are inadequate to meet energy requirements. Moreover, previous studies associated lower BMI with a poor outcome in cases of chronic heart failure^57^ ^,^ ^58^. 

Since immunosuppression may modify the natural progression of *T. cruzi* infection, some immunosuppressive conditions have previously been studied in CD patients. Systemic autoimmune diseases (SAD) are underreported in patients with *T. cruzi* infection, and the few studies that observed this association have focused on lupus erythematosus or rheumatoid arthritis possibly due to the underreporting of these conditions in low-income areas/countries^59^ ^,^ ^60^. Our SAD prevalence in patients with chronic CD of 2% was similar to the 3% recently reported by Jackson et al.^59^. A progressive increase of elderly individuals among chronic CD patients has also been observed in several endemic countries, including Brazil^6^ ^,^ ^7^ ^,^ ^61^ ^,^ ^62^. This is likely due to the decline in the number of recent infections as a consequence of vector and transfusion transmission control, as well as greater efficiency in diagnostic and therapeutic approaches^37^ ^,^ ^63^. In this study, women made up a higher proportion of the elderly CD patients^7^ ^,^ ^33^ ^,^ ^51^ ^,^ ^62^. This contrasts with other studies that report men as having the highest prevalence of CD^64^ ^-^ ^66^. This difference may be attributed to the severity of clinical forms of CD in both community-based and hospital-based study designs^50^ ^,^ ^67^. We observed a high proportion of the less severe stages (A and B) among women (64%), while the more severe stages (C and D) occurred in men (65%). Indeed, only 20% of the patients showed more severe cardiac stages, which could have influenced the final prevalence when considering sex.

Smoking and alcohol consumption are risk factors for coronary heart disease and may influence the progression of cardiac damage in patients with chronic CD^68^ ^,^ ^69^. Our results (51% and 56%, respectively) are higher than the prevalence rates of 10%^70^ for smoking and about 13%^71^ ^,^ ^72^ for alcohol consumption, reported in the Brazilian population. Previous studies found a prevalence of smoking ranging between 14 and 34%^9^ ^,^ ^10^ ^,^ ^33^ among CD patients, which are lower than our results. Although it is known that smoking may enhance the ongoing inflammatory process in some chronic diseases^73^, we did not find an association between CD clinical forms and smoking.

Most patients reported northern and northwestern regions of the state of Paraná as their place of birth/origin; these regions are considered endemic for CD^74^. Recently, Ferro and Silva^75^ found that some municipalities in the northwestern, northern and northeastern areas of the state of Paraná had an elevated risk of *T*. *cruzi* vector transmission possibly due to the high climatic and landscape suitability for vector occurrence. Meanwhile, Curitiba (in the southern region) is reported as having a low to medium suitability for both parameters. Additionally, the migration flow from rural (northern and northwestern regions) to urban (Curitiba) areas by people seeking economic activities, education, and better quality of life (including better living and healthcare conditions) increased the number of chronic CD patients in Curitiba^76^, reinforcing the idea that chronic CD is also present in low-endemic urban centers, as observed in Peru^77^ ^,^ ^78^, Bolivia^79^, and Argentina^80^. 

This study has some limitations. First is the large difference in the average age of patients with different clinical forms (*i.e.*, asymptomatic and symptomatic), as symptoms can develop many years after the infection; however, we adjusted age with multiple logistic regression. In addition, when groups were graded by cardiac severity, the sample sizes were small, so we grouped them as asymptomatic (A+B) and cardiac symptomatic (C+D). Additionally, this study also included hospital-convenience patients, often characterized to be older, which may increase the chances of comorbidities compared to community-based individuals. Thus, the community population with CD may not be precisely defined.

Finally, although we believe that the chronic CD patients of the state of Paraná might be representative of countries from the Southern Cone, further investigation in other regions of Brazil is desirable as the clinical forms may also be influenced by genetically diverse *T. cruzi* according to discrete typing units (DTUs)^81^. Thus, it is imperative to improve our understanding of the epidemiologic background and pathophysiologic aspects of this neglected disease. 

Our results indicate that the elderly population with CD are a vulnerable group due to the substantial risk of morbidity and mortality, including cardiovascular and infectious diseases. Therefore, public health policy should be continuously improved for better management of these patients.
